# Supplementary material for: Rhythms in Longitudinal Thalamic Recordings are Linked to Seizure Risk
Source: medRxiv. 2025 Oct 7:2025.10.03.25337281. Preprint. [Version 1] doi: 10.1101/2025.10.03.25337281 (PMC12632682; doi:10.1101/2025.10.03.25337281)
Supplement: 1 [file NIHPP2025.10.03.25337281V1-supplement-1.pdf]

medRxiv preprint doi: https://doi.org/10.1101/2025.10.03.25337281; this version posted October 7, 2025. The copyright holder for this preprint (which was not certified by peer review) is the author/funder, who has granted medRxiv a license to display the preprint in perpetuity.
It is made available under a CC-BY 4.0 International license .

Supplemental Materials

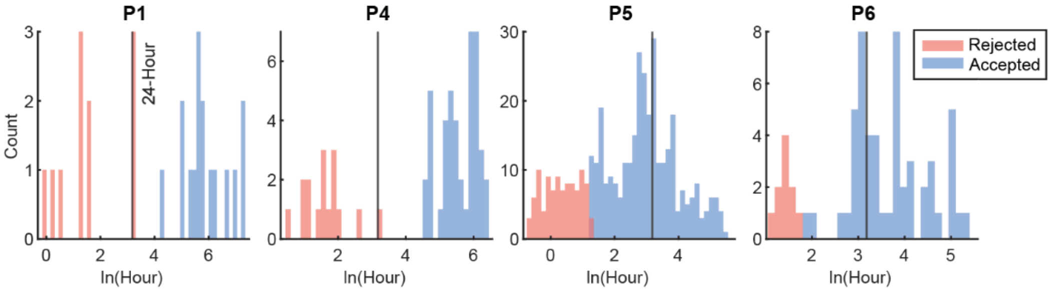

Supplementary Figure 1 - Seizure Clusters Identification

Seizures corresponding to the distribution with a shorter inter-seizure interval mean (red) were removed from the forecasting analysis. Possible seizure clusters were found in 4 participants. The x-axis shows the log-transformed inter-seizure interval. All rejected seizure events have inter-seizure intervals shorter than 24 hours.

	Left p-value	Right p-value
**P1**	**<.001**	**<.001**
P2	0.7	0.44
**P3**	**<.001**	**<.001**
**P4**	**<.001**	**<.001**
**P5**	**<.001**	**<.001**
**P6**	**<.001**	**<.001**
**P7**	**<.001**	0.85

Supplementary Table 1 - Circadian LFP Rhythm Mutual Information Permutation Test

Mutual information between the hourly mean LFP power recordings and time was compared against 1,000 random permutations of the recordings. All participants except P2 exhibited significance, indicating non-uniform circadian modulation in ANT powers.

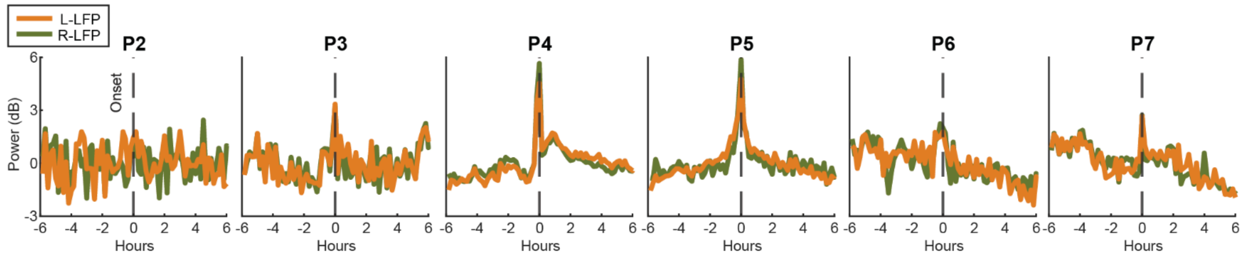

medRxiv preprint doi: https://doi.org/10.1101/2025.10.03.25337281; this version posted October 7, 2025. The copyright holder for this preprint (which was not certified by peer review) is the author/funder, who has granted medRxiv a license to display the preprint in perpetuity.
It is made available under a CC-BY 4.0 International license .

Supplementary Figure 2 - LFP Around Self-Reported Seizures

Increased theta/alpha power was found around self-reported seizures in most participants. However, the power suppression following seizures observed in P1 was not seen in other participants' recordings.

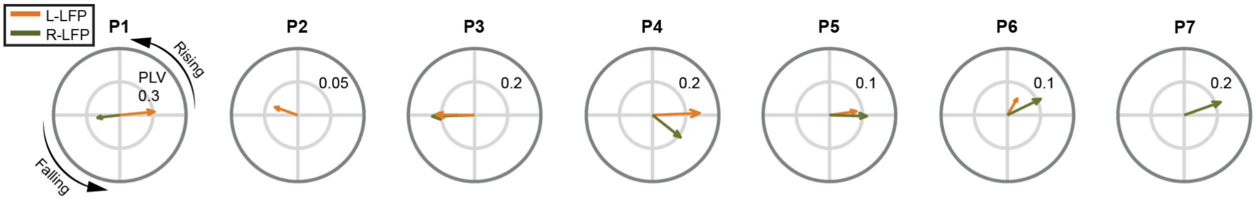

Supplementary Figure 3 - Circadian Cycles Mean Resultant Vector of Both Hemispheres

Overall seizure prevalence relative to the LFP circadian cycle phase in each hemisphere. The green and orange arrows denote the mean resultant angle and phase locking value of left and right ANT recordings, respectively. All participants, except P2 and P7, exhibited significant phase-locking in both hemispheres. Most participants had seizures clustered at a similar phase across hemispheres, but P1 exhibited opposite phase preferences between hemispheres.

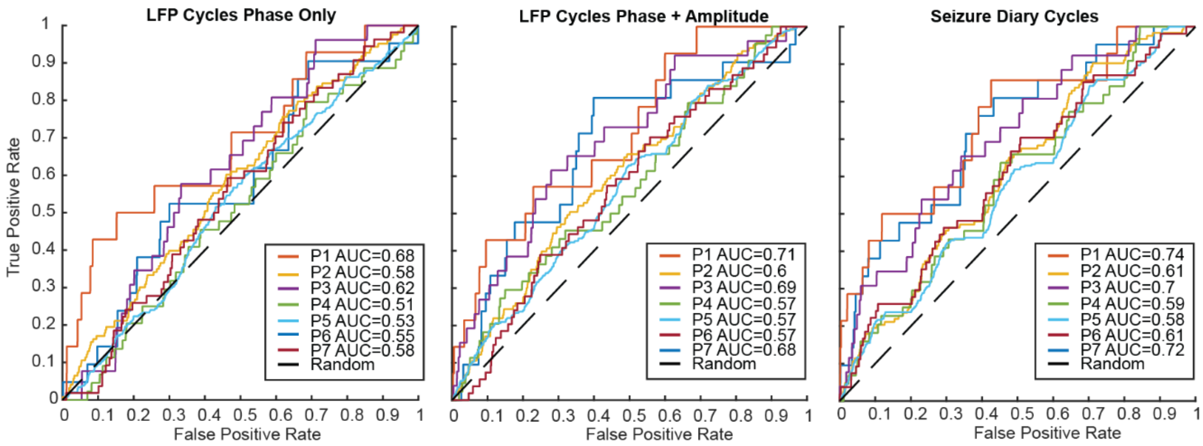

Supplementary Figure 4 - Seizure Forecasting ROCs

medRxiv preprint doi: https://doi.org/10.1101/2025.10.03.25337281; this version posted October 7, 2025. The copyright holder for this preprint (which was not certified by peer review) is the author/funder, who has granted medRxiv a license to display the preprint in perpetuity.
It is made available under a CC-BY 4.0 International license .

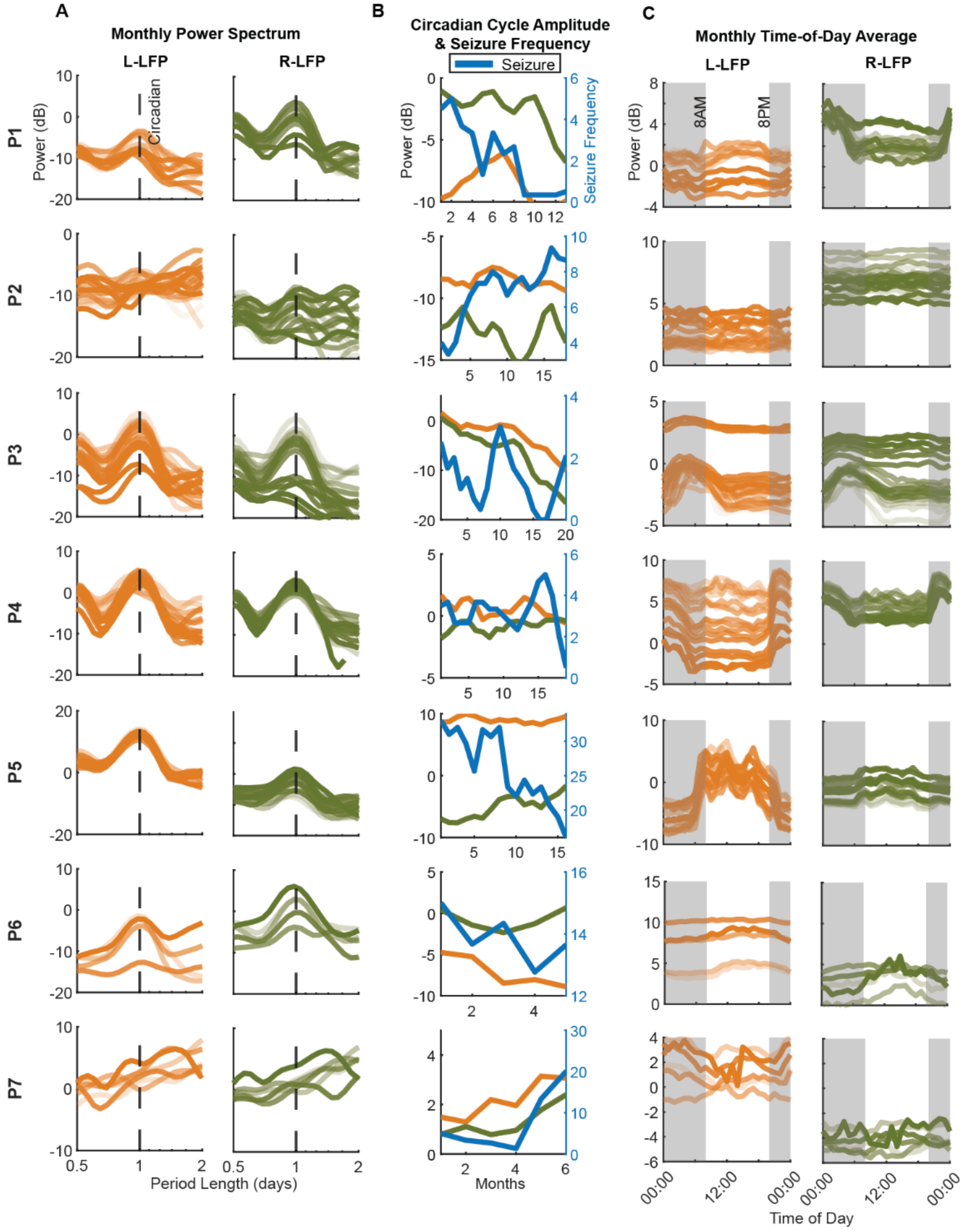

Supplementary Figure 5 - Circadian Cycle Modulation and Seizure Frequency
(A) Monthly scalograms of left and right ANT LFP powers averaged over time. Results were normalized by the impedance of recording contacts. Lines with a more transparent color indicate LFP collected earlier in the study, and vice versa. (B) 3-month moving average of circadian cycle amplitude modulation in left (orange) and right (green) hemispheres, along with seizure frequency (blue). The x-axis shows time since the start of at-home monitoring of each participant. (C) LFP averaged by time of day in each month. The same color code in (A) applies here. P1, P3, and P4 demonstrated the circadian pattern with increased activity during night (shaded in grey). Despite an impedance normalization having been applied, a potential baseline shift still exists in some participants' results.

medRxiv preprint doi: https://doi.org/10.1101/2025.10.03.25337281; this version posted October 7, 2025. The copyright holder for this preprint (which was not certified by peer review) is the author/funder, who has granted medRxiv a license to display the preprint in perpetuity.
It is made available under a CC-BY 4.0 International license .

Participant (Dominant Side)	Left ANT	Right ANT
P1 (R)	0.34	0.51
P2 (L)	-0.04	-0.15
P3 (L)	0.37	0.37
P4 (L)	0.19	-0.29
P5 (L)	0	-0.82
P6 (R)	0.48	0.22
P7 (L)	0.81	0.96

Supplementary Table 2 - Circadian Amplitude and Seizure Frequency Correlation R-value

Each row contains the 3-point moving average correlation results of LFP in each hemisphere with seizure frequency. Hemisphere with the higher circadian power is indicated in the '()' next to the participant number.

	w/ Seizure Frequency		w/ Circadian Amplitude	
	Left ANT	Right ANT	Left ANT	Right ANT
P1 (R)	-0.09	0.4	0.84	0.94
P2 (L)	0.56	-0.25	0.33	0.77
P3 (L)	0.2	0.32	0.97	0.98
P4 (L)	0.17	**0.41**	0.48	**-0.2**
P5 (L)	0.54	-0.05	0.45	0.47
P6 (L)	0.6	0.63	0.98	0.82
P7 (R)	0.46	0.91	0.83	0.91

Supplementary Table 3 - 12-hour Cycle Amplitude and Circadian Amplitude/Seizure Frequency Correlation R-value

medRxiv preprint doi: https://doi.org/10.1101/2025.10.03.25337281; this version posted October 7, 2025. The copyright holder for this preprint (which was not certified by peer review) is the author/funder, who has granted medRxiv a license to display the preprint in perpetuity.
It is made available under a CC-BY 4.0 International license .

In addition to the circadian cycle, we observed a strong 12-hour cycle in several participants' recordings. Most participants showed strong correlations between their circadian and 12-hour cycles in ANTs. However, a negative correlation was found in P4's right hemisphere (red & bold). This indicates that the circadian and 12-hour cycles could be modulated by different underlying mechanisms. A positive correlation between 12-hour cycle power and seizure frequency was found in most participants, consistent with our findings on the circadian cycle power modulation.

DominantHemisphereCircadianAmplitude ~ Time + Amps + PW + Frequency + (1|Participant)							
Variable	Estimate	SE	tStat	DF	pValue	Lower CI	Upper CI
Intercept	0.14	0.81	0.17	92	0.86	-1.47	-0.04
**Time (Month)**	-0.07	0.01	-5.76	92	**<.001**	-0.9	-0.04
Amps (mA)	1e-4	0.06	0.002	92	1	-0.11	0.11
**PW** (μs)	0.01	0.003	2.46	92	**0.02**	0.001	0.01
Frequency (Hz)	-0.001	0.005	-0.29	92	0.77	-0.01	0.008

Supplementary Table 4 - Circadian Cycle Modulation and Stimulation Parameters

Results of modeling monthly circadian cycle power and stimulation parameters using all participants' data. Significant associations between time, pulse width, and circadian power in the dominant hemisphere, with the stronger modulation over time, were found ($p<0.05$). Circadian power was z-scored within participants. A 0.01 estimate of pulse width indicates that a 1μs reduction in pulse width is associated with a 0.01 standard deviation reduction in circadian power.

Participant	Time (Month)	CircadianAmp Dominant	Seizure Frequency	Amps	PW	Freq
**1**	1	0.55	5.00	1.5	120	145
	2	0.25	6.33	1.5	120	145
	3	0.34	5.00	3	90	145
	4	0.83	4.33	3	90	145
	5	1.03	1.33	3	90	145
	6	0.78	3.33	3	90	145
	7	-0.14	2.33	3	90	145
	8	-0.49	3.00	4.3	50	125
	9	-0.25	1.00	4.3	50	125

medRxiv preprint doi: https://doi.org/10.1101/2025.10.03.25337281; this version posted October 7, 2025. The copyright holder for this preprint (which was not certified by peer review) is the author/funder, who has granted medRxiv a license to display the preprint in perpetuity.
It is made available under a CC-BY 4.0 International license .

	10	-0.09	1.00	4.3	50	125
	11	-0.40	1.00	3	90	145
	12	-1.09	0.67	4.3	50	125
	13	-1.44	1.00	4.3	50	125
**2**	1	0.37	4.00	4	90	145
	2	0.05	3.33	4	90	145
	3	0.02	4.00	5	60	125
	4	0.03	5.67	5	60	125
	5	0.33	6.67	4	90	145
	6	1.07	7.34	4	90	145
	7	1.62	7.34	4	90	145
	8	1.57	8.00	4	90	145
	9	0.60	7.67	4	90	145
	10	-0.48	6.67	4	90	145
	11	-0.78	7.34	4	90	145
	12	-0.81	7.67	4	90	145
	13	-0.64	7.00	4	90	145
	14	-0.48	7.34	4	90	145
	15	-0.23	8.00	4	90	145
	16	-0.38	9.34	4	90	145
	17	-0.69	8.76	4	90	145
	18	-0.96	8.64	4	90	145
**3**	1	1.92	2.50	2	90	145
	2	1.39	1.67	3	90	145
	3	1.28	2.00	3	90	145
	4	0.67	1.00	1.7	140	165
	5	0.97	1.33	1.7	140	165
	6	0.19	0.67	1.7	140	165
	7	0.01	0.33	1.7	140	165
	8	-0.27	1.00	1.7	140	165
	9	0.00	2.33	1.7	140	165
	10	0.14	3.00	1.7	140	165
	11	0.00	2.33	2.9	60	165
	12	-0.02	1.67	2.9	60	165
	13	-0.35	1.33	2.9	60	165

medRxiv preprint doi: https://doi.org/10.1101/2025.10.03.25337281; this version posted October 7, 2025. The copyright holder for this preprint (which was not certified by peer review) is the author/funder, who has granted medRxiv a license to display the preprint in perpetuity.
It is made available under a CC-BY 4.0 International license .

	14	-0.68	1.00	2.9	60	165
	15	-0.98	0.33	2.9	60	165
	16	-1.07	0.00	2.9	60	165
	17	-1.18	0.00	2.9	60	165
	18	-1.11	0.67	2.9	60	165
	19	-0.67	1.38	2.9	60	165
	20	-0.35	2.07	2.9	60	165
**4**	1	0.61	3.50	2.7	90	125
	2	0.26	3.67	2.7	90	125
	3	0.09	2.67	2.7	90	125
	4	1.33	2.67	2.7	90	125
	5	1.45	2.67	2.7	90	125
	6	1.16	3.67	2.5	90	145
	7	0.02	3.67	2.5	90	145
	8	-0.09	3.33	2.7	90	125
	9	-0.28	3.33	2.7	90	125
	10	-0.45	3.00	2.7	90	125
	11	-0.46	2.67	2.7	90	125
	12	-0.47	2.33	2.7	90	125
	13	-0.47	3.00	3.3	60	125
	14	-0.48	3.67	3.3	60	125
	15	-0.49	4.67	2.7	90	125
	16	-0.50	5.00	2.7	90	125
	17	-0.51	4.00	2.7	90	125
	18	-0.51	2.00	2.7	90	125
	19	-0.51	0.50	2.7	90	125
**5**	1	-0.53	66.52	4.5	90	145
	2	-0.51	62.35	5.9	120	125
	3	-0.22	60.35	4.5	90	145
	4	0.89	44.34	5.9	120	125
	5	1.47	36.34	5.9	120	125
	6	1.64	50.01	4.2	120	125
	7	0.42	46.01	5.9	60	125
	8	-0.24	45.34	5.9	60	125
	9	-0.80	32.34	5.9	60	125

medRxiv preprint doi: https://doi.org/10.1101/2025.10.03.25337281; this version posted October 7, 2025. The copyright holder for this preprint (which was not certified by peer review) is the author/funder, who has granted medRxiv a license to display the preprint in perpetuity.
It is made available under a CC-BY 4.0 International license .

	10	-0.80	32.01	5.9	60	125
	11	-0.70	31.01	4.2	120	125
	12	-0.14	25.67	4.2	120	125
	13	0.11	23.01	4.2	120	125
	14	0.10	20.67	4.8	90	125
	15	-0.29	19.00	4.8	90	125
	16	-0.41	16.00	4.2	120	125
**6**	1	-0.65	15.00	4	90	145
	2	-0.39	13.67	4	90	145
	3	-0.34	14.34	4	90	145
	4	0.43	12.77	4	90	145
	5	0.58	13.65	4	90	145
**7**	1	-0.27	5.00	1	90	145
	2	-0.52	3.33	1	90	145
	3	-0.42	2.67	1	90	145
	4	-0.46	1.33	1	90	145
	5	0.52	13.35	1	90	145
	6	0.87	20.02	1	90	145

Supplementary Table 5 - Participant Deep Brain Stimulation Parameters
The dominant hemisphere was selected based on which hemisphere exhibited a stronger circadian cycle across time. Circadian amplitude was z-scored.
